# Vitamin B6 Deficiency Promotes Loss of Heterozygosity (LOH) at the *Drosophila* *warts* (*wts*) Locus

**DOI:** 10.3390/ijms23116087

**Published:** 2022-05-29

**Authors:** Eleonora Gnocchini, Eleonora Pilesi, Ludovica Schiano, Fiammetta Vernì

**Affiliations:** Department of Biology and Biotechnology “Charles Darwin”, Sapienza University, 00185 Rome, Italy; gnocchini.1657027@studenti.uniroma1.it (E.G.); eleonora.pilesi@uniroma1.it (E.P.); schiano.1916276@studenti.uniroma1.it (L.S.)

**Keywords:** pyridoxal 5′ phosphate (PLP), vitamin B6, loss of heterozygosity (LOH), mitotic recombination, *warts*, *Drosophila*

## Abstract

The active form of vitamin B6, pyridoxal 5′-phosphate (PLP), is a cofactor for more than 200 enzymes involved in many metabolic pathways. Moreover, PLP has antioxidant properties and quenches the reactive oxygen species (ROS). Accordingly, PLP deficiency causes chromosome aberrations in *Drosophila*, yeast, and human cells. In this work, we investigated whether PLP depletion can also cause loss of heterozygosity (LOH) of the tumor suppressor *warts* (*wts*) in *Drosophila*. LOH is usually initiated by DNA breakage in heterozygous cells for a tumor suppressor mutation and can contribute to oncogenesis inducing the loss of the wild-type allele. LOH at the *wts* locus results in epithelial *wts* homozygous tumors easily detectable on adult fly cuticle. Here, we found that PLP depletion, induced by two PLP inhibitors, promotes LOH of *wts* locus producing significant frequencies of *wts* tumors (~7% vs. 2.3%). In addition, we identified the mitotic recombination as a possible mechanism through which PLP deficiency induces LOH. Moreover, LOH of *wts* locus, induced by PLP inhibitors, was rescued by PLP supplementation. These data further confirm the role of PLP in genome integrity maintenance and indicate that vitamin B6 deficiency may impact on cancer also by promoting LOH.

## 1. Introduction

Pyridoxal 5′-phosphate (PLP), the active form of vitamin B6, is involved in a wide range of metabolic processes. It is the cofactor of about 200 enzymes that perform reactions such as transaminations, decarboxylations, racemizations, and β/γ-eliminations. Most of these reactions are related to amino acid biosynthesis and degradation, but vitamin B6 is also involved in other processes, including sugar and fatty acid metabolism [1]. In addition, PLP is involved in the synthesis and catabolism of neurotransmitters such as serotonin, histamine, and g-aminobutyric acid (GABA) [2] and in the regulation of the immune system, although this latter role has been less explored [3]. Vitamin B6 works in the *one-carbon metabolism*, a pathway that enables cells to generate one-carbon units (also referred to as methyl groups) to be utilized for nucleic acid synthesis and DNA methylation processes [4]. In particular, in this pathway, vitamin B6 is a cofactor for serine hydroxymethyltransferase (SHMT), the enzyme that produces 5,10-methylene THF, the methyl donor used afterwards by thymidylate synthase to produce dTMP from dUMP [5]. Thus, is it easy to predict that impairment of this function has a strong impact on DNA integrity. Vitamin B6 protects the genome also by working as an antioxidant molecule able to counteract reactive oxygen species (ROS) and advanced glycation end-products (AGEs) [6]. In animal cells, PLP is synthesized from precursors taken from food such as pyridoxal (PL), pyridoxamine (PM), and pyridoxine (PN) thanks to the activity of pyridoxal 5′-phosphate kinase (PDXK) and pyridoxine pyridoxamine 5′-phosphate oxidase (PNPO) enzymes [6]. Vitamin B6 is present in many different foods; thus, severe deficiencies are rather rare in developed countries. However, many diseases including cancer, metabolic conditions, and drugs have been associated with reduced PLP levels, although underlying mechanisms remain almost unknown. In particular, PLP concentrations tend to be low in people with alcohol dependence, obese individuals, and pregnant women, as well as in people affected by chronic renal insufficiency, kidney diseases, and malabsorption syndromes such as celiac disease and inflammatory bowel diseases. Moreover, the assumption of some antibiotics or antidepressant drugs may reduce PLP availability [6]. Regarding the relationship between vitamin B6 and cancer, many studies have indicated an inverse relationship between PLP plasmatic levels and several cancers, including colorectal and lung cancers [7,8,9], while, on the other hand, other studies have demonstrated that vitamin B6 depletion is beneficial for some hematic cancers [10]. We and others have demonstrated that reduced PLP levels, induced by mutations in genes involved in PLP synthesis (*dPdxk* or *Sgll/PNPO*) or by PLP inhibitors (such as isoniazid, penicillamine, 4 deoxypyridoxine (4DP)), cause DNA damage in yeast and flies, resulting in an elevated frequency of chromosome aberrations (CABs), which are rescued by PLP administration [11,12,13]. Similar results were also found in human cells by confirming that PLP protects the human genome from damage [12]. We demonstrated that reduced PLP levels in *Drosophila*, in addition to CABs, cause diabetes and that hyperglycemia is largely responsible for DNA damage [12,13]. Interestingly, we found that low PLP levels synergize with high glucose levels by exacerbating chromosome damage; this finding raised the hypothesis that in diabetic patients, PLP levels need to be kept under control to reduce cancer risk [14]. Furthermore, we found that individuals bearing mutations in the *Drosophila dPdxk* gene display an increased dUTP/dTTP ratio compared to controls, suggesting that, although to a lesser extent, chromosome damage can also depend upon the PLP role as a cofactor in *one-carbon* metabolism [12]. Taken together, these findings allowed us to hypothesize that one of the ways through which PLP can impact on cancer is by impairing DNA integrity.

Here, starting from the evidence that PLP deficiency causes DNA breakage, we wanted to investigate whether reduced levels of this vitamin could cause loss of heterozygosity (LOH).

LOH occurs when diploid cells, which are heterozygous for a mutant allele, lose the wild-type copy of the gene and express the mutant phenotype. LOH is mainly caused by chromosome deletions or mitotic recombination, and by segregation defects, although to a lesser extent. [15]. Interestingly, LOH has been detected in several tumor suppressor genes and has been associated with different kinds of tumors; thus, the opportunity of exploiting the differences between tumor and normal cells has been glimpsed as possible therapy for a precision medicine approach [16,17,18,19,20]. A classic example of LOH is that occurring at the *Retinoblastoma* gene (*Rb*). In cells in which the first *Rb* allele is lost by random mutation, it has been demonstrated that the LOH phenomenon is at the basis of the loss of the second allele [21]. Following this finding, several candidate genes were discovered as tumor suppressors by characterizing sites of prevalent LOH in human cancers [22]. The tumor suppressors more frequently involved in LOH and hence in cancer are *TP53*, *Phosphatase* and *tensin homolog (PTEN)*, and *adenomatous polyposis coli (APC)* genes [23,24,25]. In addition, LOH is particularly relevant in inherited cancer predisposition syndromes, which present germline mutations in tumor suppressors such as *BRCA1* and BRCA2 and play a crucial role in increasing the cancer risk [26].

In *Drosophila*, the discovery that recombination can take place also in mitosis and give rise to LOH provided a powerful method to generate mosaics, which have been extensively exploited by developmental biologists [27]. In addition, the somatic clones have been exploited in screenings aimed at identifying genes involved in DNA repair [28]. Moreover, LOH became a compelling strategy to generate *Drosophila* cancer models [29]. The *multiple wing hair* (*mhw*) wing-spot test and the *white* test, known as Somatic Mutation And Recombination Tests (SMARTs), were extensively employed to assess the genotoxicity of several compounds in *Drosophila* somatic cells [30,31].

More recently, a new SMART test based on LOH occurring at the tumor suppressor *warts* (*wts*) gene has been developed [32,33]. In the *wts* system, LOH arises in cells of larval imaginal discs, which represent the primordia of adult epithelial structures, and produces homozygous *wts* cells, which appear as tumors located on several body parts of adult flies. *warts* encodes a serine/threonine kinase working in the *hippo* pathway [34,35] and its human counterpart, *large tumor suppressor kinase 1 (LATS1)*), is a tumor suppressor involved in the negative regulation of CDC2 kinase activity [36,37]. *LATS^−/−^* mice develop soft-tissue sarcomas and ovarian stromal cell tumors and are highly sensitive to carcinogenic treatments [38].

Given that LOH is often initiated by a DNA breakage event, here, we wanted to test whether PLP inhibitors could lead to the development of *wts* tumors, consistent with the impact of PLP deficiency on DNA damage. The rationale of this work was to further reinforce the involvement of PLP in DNA damage and in addition, by translating the results to humans, to draw attention to the potential role of vitamin B6 depletion in the LOH phenomenon associated with cancer.

## 2. Results

### 2.1. PLP Depletion Causes Loss of Heterozygosity (LOH)

**Figure 1:**
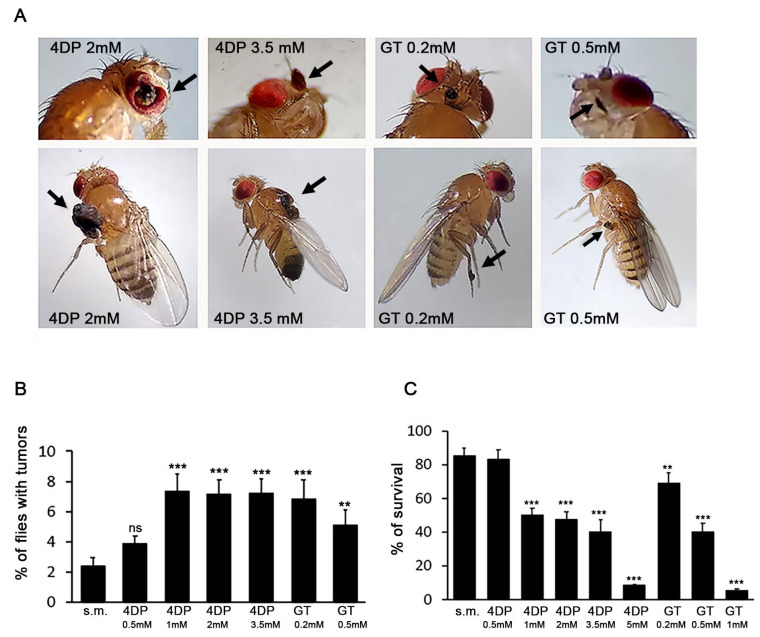
*PLP inhibitors induce wts tumors*. (**A**) Examples of *wts* tumors in *wts/+* flies treated with different concentrations of PLP inhibitors (arrowed). (**B**) Quantification of the results. Each column represents the mean value ± SEM. For each condition, 300–700 flies from three different experiments were examined. (**C**) Percentage of flies reaching the adult stage. Each column represents the mean value ± SEM. For each condition, about 500 flies from three different experiments were examined. s.m. = standard medium; 4DP = 4-deoxypyiridoxine; GT = ginkgotoxin; ns = non-significant Chi-square test. **, *** significant Chi-square test with *p* < 0.01 and <0.001, respectively.

To investigate whether PLP depletion may promote LOH at the *warts* (*wts*) locus located near the tip of the right arm of chromosome III [35], we mated wild-type females (+/+) to *wts/TM3* males on growth media containing the PLP inhibitor 4-deoxypyridoxine (4DP) and examined the *wts/+* F1 progeny for the presence of *wts* homozygous tumor clones on the adult cuticle. We tested four different 4DP concentrations (0.5–3.5 mM) chosen on the basis that 2 mM concentrations cause CABs in *Drosophila* neuroblasts [14]. 4DP concentrations ≥ 1 mM produced about three times more *wts* tumors than the standard medium (5.9–7.2% vs. 2.35%) (Figure 1A,B). No *wts* tumors were found in untreated or 4DP-treated *TM3/+* siblings. We also performed the reciprocal cross (*wts/TM3* females x wild-type males), finding similar results, so we unified the data. Concentrations higher than 3.5 mM in the growth medium induced high toxicity allowing only 8% of laid eggs to develop into adults (Figure 1C), thus preventing the analysis of tumors. To confirm the validity of our approach, we also tested the effect of X-rays (3.5Gy) on *wts/+* flies, finding 17% of flies with *wts* tumors according to (Figure S1) [33]. To corroborate the effects produced by 4DP, we also tested another PLP inhibitor, the ginkgotoxin (GT). This drug has never been used before in *Drosophila*; thus, we tested the concentrations employed in zebrafish larvae [39]. As shown in Figure 1A,B, 0.2 mM GT produced 6.8% of flies with tumors, whereas 0.5 mM GT induced *wts* tumors only in ~5% of flies, probably due to the toxicity of the drug (Figure 1C). GT concentrations higher than 0.5 mM killed most of the eggs or larvae (5% survival) and were not tested (Figure 1C).

Tumors induced by PLP inhibitors mainly arose on the notum and wings. However, we found tumors also in other body parts such as the eyes, head, halteres, or legs on which spontaneous tumors are less frequent (Table 1) [32,33].

### 2.2. PLP Supplementation Rescues LOH at wts Locus

**Figure 2:**
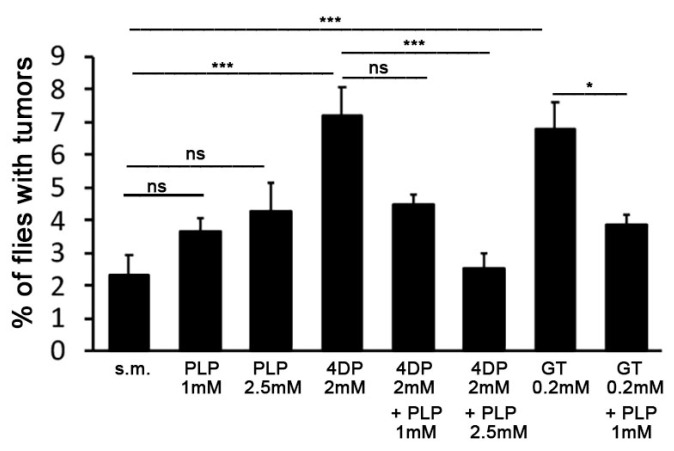
*PLP supplementation rescues wts tumors.* Each column represents the mean value ± SEM. For each condition, at least 500 flies were examined in three different experiments. *, *** significant Chi-square test with *p* < 0.05 and <0.001, respectively. ns = non-significant Chi-square test; s.m. = standard medium; PLP = pyridoxal 5′-phosphate; 4DP = 4-deoxypyridoxine; GT = ginkgotoxin.

To confirm that *wts* tumors in 4DP- and GT-treated flies were caused by PLP deficiency, we added PLP to media containing the inhibitors. We first tested the effect of PLP on the standard medium finding a slight but non-significant increase in the frequency of flies with tumors with respect to the untreated controls. The frequency of flies with tumors, grown in 2 mM 4DP plus 1 mM PLP, resulted decreased (4.6%) with respect to that found in flies grown in 4DP (~7%), but this reduction was not statistically significant (Figure 2). In contrast, 2 mM 4DP plus 2.5 mM PLP treatment significantly decreased tumor appearance (2.55%). The 1 mM PLP concentration was instead capable of significantly reducing the frequency of tumors induced by 0.2 mM GT (Figure 2). As reported in Table 2, PLP treatment did not affect the tumor distribution that was mainly observed on the wings and notum.

### 2.3. Mitotic Recombination as a Possible Mechanism of LOH at wts Locus

LOH can be produced by chromosome deletion, chromosome loss, mitotic recombination, and chromosome segregation errors [21]; between these mechanisms, mitotic recombination is the most frequent. To investigate whether the somatic recombination could be also at the basis of *wts* tumors induced by 4DP, we examined the appearance of *wts* tumors on the body of *wts/TM3* flies in which the presence of multiply inverted third chromosome balancer *TM3* suppresses the mitotic recombination [40].

**Figure 3:**
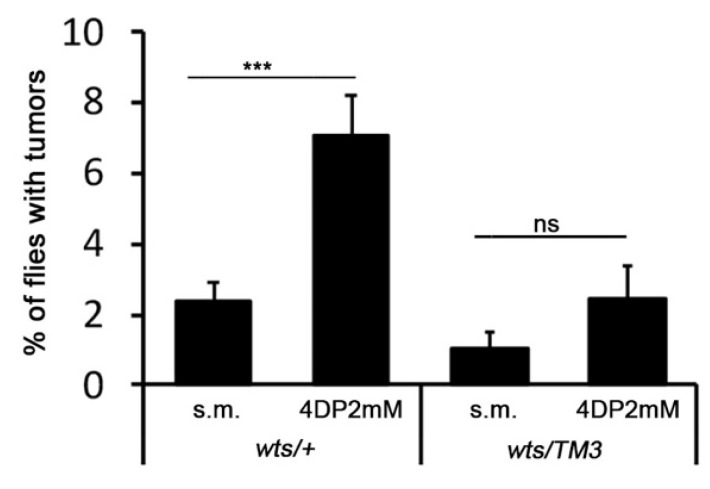
*Mitotic recombination as a possible mechanism of LOH.* The *TM3* balancer chromosome prevents LOH. Each column represents the mean value ± SEM. For each condition, more than 500 flies were examined in three different experiments. *** significant Chi-square test with *p* < 0.001; ns = non-significant Chi-square test. s.m. = standard medium; 4DP = 4 deoxypyridoxine.

As shown in Figure 3, we found a strong reduction in tumor frequency in 4DP-fed flies, suggesting that mitotic recombination may be a possible mechanism through which PLP deficiency promotes *wts* tumors.

### 2.4. Effect of High Sugar Diet on wts Tumors Induced by PLP Deficiency

**Figure 4:**
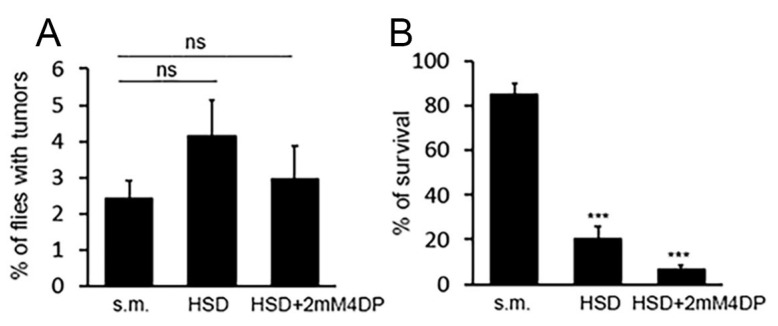
*Effect of the combined treatment HSD + 4DP on LOH at the wts locus*. (**A**) Quantification of tumors in *wts/+* flies reared in the indicated media. For each condition, more than 500 flies were examined in three different experiments. Each column represents the mean value ± SEM. (**B**) Percentage of flies that reach the adult stage. Each column represents the mean value ± SEM obtained by examining about 500 flies per condition in three different experiments. s.m. = standard medium HSD = high sugar diet; 4DP = 4 deoxypyridoxine; ns = non-significant Chi-square test. *** Significantly different in Chi-square test, with *p* < 0.001.

We previously demonstrated a synergistic effect between low PLP levels and high sugar levels in the induction of CABs [14]. Thus, here, we tested whether a sugar-rich diet, (HSD, a standard medium containing 1M sucrose instead of 0.15M) combined with 4DP could enhance *wts* tumor formation in *wts/+* flies. We found that HSD alone did not show any significant effect (~4% of flies with tumors) (Figure 4A), according to the finding that this treatment does not produce CABs [12]. Surprisingly, even the combined treatment HSD plus 2 mM 4DP did not produce any effect (2.97% of flies with tumors) (Figure 4A). However, we found that HSD treatment alone reduced the survival to 20%, and the combined treatment (HSD plus 2 mM 4DP) reduced the survival to 6.5% (Figure 4B). However, given that *TM3/+* siblings coming from the same cross (*wts/TM3* × *+/+*) displayed the same percentage of survival than *wts/+* flies, the lethality of *wts/+* flies cannot be attributed only to an increased frequency of tumors.

In conclusion, our data indicate that vitamin B6 deficiency can promote LOH possibly through mitotic recombination and suggest that LOH is another potential mechanism, besides DNA damage, through which the deficiency of this vitamin could impact on cancer.

## 3. Discussion

In this work, we demonstrated that PLP deficiency can cause LOH at the *wts* gene of *Drosophila*, hence inducing the development of epithelial tumors. In contrast, we showed that the administration of PLP, together with PLP inhibitors such as 4DP or GT, prevents the appearance of tumors. The relation between vitamins, DNA damage, and cancer is attested by a growing number of studies, and it is widely accepted that sub-optimal levels of key micronutrients required for DNA maintenance reduce genomic stability, producing similar effects to inherited genetic disorders or exposure to carcinogens [41,42]. Consistently, we and others have demonstrated that reduced levels of vitamin B6 in *Drosophila*, yeast, and human cells cause CABs due to antioxidant properties of PLP and/or to the role of PLP in *one-carbon metabolism* [11,12]. These findings suggest that PLP deficiency can promote cancer by increasing the frequency of CABs, which are strictly associated with the tumorigenesis process [43].

Another genetic event commonly associated with cancer is LOH occurring at loci where tumor suppressor genes map [21]; in heterozygous cells carrying a mutation in a tumor suppressor gene, the loss of the wild-type allele is indeed responsible for cancer phenotype expression [44]. Given that the more frequent mechanisms at the basis of LOH, such as chromosome deletions and mitotic recombination, both initiate with DNA breakage events, it is conceivable to suppose that any event able to cause DNA breakage can potentially cause LOH. However, although many papers have reported a correlation between reduced micronutrient levels and cancer, there are no studies focused on the impact of reduced vitamin levels on LOH, probably due to difficulties to perform such a study on humans. Thus, our study in *Drosophila* is the first in which vitamin deficiency and LOH have been correlated. In this paper, we used the *wts* SMART test to further confirm the genotoxicity of PLP deficiency. The *wts* test has been proven to be highly sensitive because besides confirming the genotoxicity of drugs previously assessed in other tests. It allowed revealing the genotoxicity of compounds known to be genotoxic in humans but did not show any effect on other *Drosophila* SMART systems [32]. Here, we showed that two different PLP inhibitors, 4DP and GT, were both able to induce tumors on *wts/+* flies, although without dose-dependent effects. An explanation can be that we used a narrow interval of concentrations due to the toxicity of both compounds, which strongly reduced fly vitality. We were not able to examine the effects of 4DP concentrations higher than 3.5 mM or GT concentrations higher than 0.5 mM due to the high mortality of the progeny. The frequency of *wts* tumors on 4DP-fed flies was very similar to that of flies grown on GT-supplemented medium, probably because both drugs inhibit PLP synthesis with the same mechanisms, consisting in a competitive inhibition with PLP precursors at the active site of the PDXK enzyme [45].

We found that PLP treatment combined with the PLP inhibitors prevented the formation of *wts* tumors. However, adding PLP to the standard medium increased the frequency of tumors with respect to the untreated controls, although not in a statistically significant way. To explain this apparent contradiction, we can hypothesize that while on the one hand, PLP deficiency induces *wts* tumors by causing LOH, on the other hand, in cells in which LOH occurs spontaneously, PLP supplementation may promote further cell growth, thus leading to the tumor becoming visible.

Both 4DP and GT treatments resulted three times more tumorigenic than the standard diet. Although this effect is low if compared with that elicited by strong mutagens such as X rays (which we used as the positive control) or the methyl methanesulfonate (MMS) [33], it was comparable to that produced by less strong carcinogens such as benzo(e)pyrene, aflatoxin AFB2, 4-acetylaminofluorene (4-AAF), 4-(methylnitrosamino)-1-(3-pyridyl)-1-butanone (NNK), and oxoplatin on the same system [32].

With respect to other SMART tests, *wts* gives the possibility of studying the different distribution of tumors on different tissues. *wts* tumors have been found not homogeneously distributed over the cuticle [32]. This depends upon the final organ size and upon the period of cell proliferation. Consistently, notum and wings are the most affected. Tumors in body parts with a short proliferation period such as the eye and leg discs are less frequent. The majority of tumors induced by PLP inhibitors are located on the same body parts where they occur in flies grown on standard medium. However, flies also grown on PLP inhibitor-supplemented media displayed more frequent tumors on body parts, resulting less affected in flies grown on standard medium (i.e., eyes and legs), although the number of the examined control flies was higher. We can hypothesize that spontaneous tumors can also occur on the eyes or legs, but they do not reach the dimensions that allow them to be detected. In contrast, PLP deficiency might alter the metabolism of *wts* homozygous cells, thus leading them to proliferate at a higher rate and become visible.

We found that PLP inhibitors did not produce a significant frequency of tumors on the body of *wts/TM3* flies. As the balancer chromosomes prevent recombination also in mitosis, this finding indicates that PLP deficiency induces LOH by promoting mitotic recombination [40]. By considering that mitotic recombination results from the breakage and reunion of chromatids in the four-strand stage, these data further confirm the role of PLP in DNA integrity maintenance. In addition, our findings are consistent with those of Smith et al. [28], showing a clear correspondence between chromosome damage and mitotic recombination. In particular, these authors showed that the depletion of essential mitotic functions involved in chromosome integrity maintenance also increased the frequency of *mwh* somatic clones produced by mitotic recombination [28].

We previously demonstrated that PLP deficiency synergizes with sugars to promote DNA damage [12], suggesting that reduced PLP levels can represent a cancer risk factor in diabetic patients [14]. However, although we found a drastic reduction in survival (6.5%) in *wts/+* flies (as well as in their *TM3*/+ siblings) reared on HSD + 2 mM 4DP medium, we found a frequency of tumors not different from controls. To explain this, we can hypothesize that most of the flies died during the high toxicity of the treatment, and only healthy flies managed to survive. We previously demonstrated that 2 mM 4DP treatment causes 10–15% of CABs in wild-type flies, whereas HSD + 2 mM 4DP treatment causes 60% of CABs in neuroblasts [14]. Given that LOH is initiated by DNA breakage, we can envisage that HSD + 2 mM 4DP treatment might produce 4–5-fold more events of LOH at the *wts* locus with respect to 4DP alone. Thus, it is reasonable to expect that larvae with 60% of CABs and possibly more than one tumor can hardly reach the adult stage. However, although from our results it is not possible to conclude that a PLP deficiency combined with sugars increases LOH frequency, based on the strong synergistic effect of the two treatments on CABs, we are confident that this relationship can be demonstrated in the future in other systems or using different strategies.

In conclusion, our results, translated to humans, indicate that PLP deficiency not only impacts on cancer by promoting CABs but also by promoting LOH. In particular, decreased PLP levels may increase cancer susceptibility by stimulating LOH when a muted copy of a tumor suppressor gene, such as *BRCA1/2* or *Rb1* is inherited in the germline. In addition, our study may stimulate further investigations concerning the impact of other micronutrients on LOH.

## 4. Materials and Methods

### 4.1. Drosophila Stocks and Genetic Crosses

The EMS-induced *wts*^3–17^ allele was obtained from Bloomington Stock Center (Bl No. 7052). Oregon R strain was used as a wild-type control. The balancers used in this work and the genetic markers are described in detail on FlyBase (http://flybase.bio.indiana.edu/ (accessed on 26 May 2022)).

All stocks were maintained and crossed at 25 °C on a standard medium containing (in 100 mL): agar (0.68 g), yeast (6.52 g), flour (3 g), propionic acid (600 μL), and sucrose (5.13 g = 0.15 M for standard food; 34.2 g = 1.0 M for high sugar diet (HSD)).

Genetic crosses were performed by mating Oregon R virgin females to *wts/TM3* males in standard media, media containing PLP inhibitors, or supplemented with PLP and the *wts/+* or *TM3/+* progeny was scored for tumors.

### 4.2. Analysis of Flies with Tumors and Survival Evaluation

The analysis of tumors was made in blinded conditions by counting from 300 to 700 flies depending on the recovery of the progeny due to the toxicity of treatments.

Pictures of adult flies were taken using a Nikon D5200 digital camera mounted on a stereomicroscope (Nikon SMZ-1). Pictures were taken using a 1/6 s exposure and 800 iso.

The toxic effect of the tested substances was measured as survival percentage, i.e., the percent of emerged flies from the number of eggs laid during 24 h of egg laying.

### 4.3. Treatments

PLP inhibitors such as 4DP (Sigma Cat. No. D0501) and GT (Sigma Cat. No. 89960), as well as PLP (Sigma Cat. No. P9255), were dissolved in the standard medium at the concentrations indicated in the results. We tested four different 4DP concentrations (0.5–3.5 mM) chosen on the effect of this drug in a previous experiment performed on *Drosophila* neuroblasts [14]. As GT has never been used before in *Drosophila*, we used concentrations employed in zebrafish larvae [39].

Alternatively, the larvae were irradiated in the culture vials with 3.5 Gy of X-rays generated by an MLG 300/6 Gilardoni device.

We used protocols according to ethical approval.

### 4.4. Statistical Analysis

To evaluate the statistical significance of the results we used the Chi-square test (2 × 2 table). *, **, and *** refer to *p* values < 0.05, 0.01 and 0.001, respectively.

## Figures and Tables

**Table 1:** Distribution of wts tumors induced by PLP inhibitors on wts/+ flies.

Treatment	Tot. Flies	N. of Tumors	Eye	Head	Wing	Body	Leg	Halter
s.m.	724	17	0.00%	6.06%	45.45%	48.48%	0.00%	0.00%
4DP 0.5 mM	568	22	0.00%	4.55%	68.18%	22.73%	4.55%	0.00%
4DP 1 mM	301	24	0.00%	4.17%	62.50%	29.16%	4.17%	0.00%
4DP 2 mM	323	22	9.09%	18.18%	31.82%	36.36%	4.55%	0.00%
4DP 3.5 mM	320	24	4.17%	4.17%	45.83%	45.83%	0.00%	0.00%
GK 0.2 mM	428	30	3.33%	6.67%	46.67%	30.00%	6.67%	6.67%
GK 0.5 mM	508	29	3.45%	6.90%	48.28%	37.93%	0.00%	3.45%

Values represent the percentage distribution of tumors scored in the indicated parts of body.

**Table 2:** Distribution of *wts* tumors on the body of *wts/+* flies reared in PLP-supplemented medium.

Treatment	Tot. Flies	N. of Tumors	Eye	Head	Wing	Body	Leg	Halter
s.m.	724	17	0.00%	6.06%	45.45%	48.48%	0.00%	0.00%
PLP 1 mM	408	15	0.00%	6.67%	66.67%	20.00%	6.67%	0.00%
PLP 2.5 mM	726	37	0.00%	13.51%	35.14%	45.95%	2.70%	2.70%
4DP 2 mM	323	22	9.09%	18.18%	31.82%	36.36%	4.55%	0.00%
4DP 2 mM + PLP 1 mM	506	25	4.00%	16.00%	40.00%	36.00%	4.00%	0.00%
GK 0.2 mM	428	30	3.33%	6.67%	46.67%	30.00%	6.67%	6.67%
GK 0.2 mM + PLP 1 mM	468	19	0.00%	26.32%	26.32%	47.37%	0.00%	0.00%

Values represent the percentage distribution of tumors scored in the indicated parts of body.

## Data Availability

Not applicable.

## Supplementary Materials

The following supporting information can be downloaded at: https://www.mdpi.com/article/10.3390/ijms23116087/s1.
