# Supplementary material for: Vitamin B6 Deficiency Promotes Loss of Heterozygosity (LOH) at the Drosophila warts (wts) Locus
Source: Int J Mol Sci. 2022 May 29;23(11):6087. doi: 10.3390/ijms23116087 (PMC9181336; doi:10.3390/ijms23116087)
Supplement: Supplementary file 1 [file ijms-23-06087-s001.zip › ijms-1741885-supplementary.pdf]

Supplementary figure

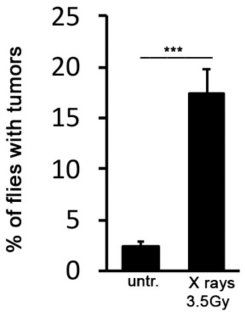

Figure S1. *X-rays induce LOH at wts locus*. Quantification of tumors displayed from *wts*/+ flies grown in standard medium untreated or X-rays treated. Columns indicate the mean value ± SEM from three different experiments in which have been examined about 300 flies per condition. Untr.= untreated *** Significantly different in Chi square test, with $p < 0.001$.
